# Clinical Correlates of Hachinski Ischemic Score and Vascular Factors in Cognitive Function of Elderly

**DOI:** 10.1155/2014/852784

**Published:** 2014-08-26

**Authors:** Youn Ho Kim, Oh Dae Kwon

**Affiliations:** Daegu Catholic University Medical Center, Department of Neurology, School of Medicine, Catholic University of Daegu, 3056-6 Daemyeong 4-dong, Nam-gu, Daegu 705-718, Republic of Korea

## Abstract

The aim of this study is to investigate the relationship between Hachinski ischemic score (HIS) and vascular factors as well as between HIS and the cognitive function in elderly community. Demographic characteristics, such as sex, age, education, history of drinking and smoking, family history of dementia and stroke, diabetes mellitus, hypertension, hyperlipidemia, cardiovascular disease, stroke, and dementia, were surveyed. Neurological examination was administered to every subject and HIS was checked by a neurologist. From a total of 392 participants aged 65 and over in a rural community, 348 completed the survey and were finally enrolled. Among the vascular factors, history of hypertension (*P* = 0.008), history of stroke (*P* < 0.001), family history of dementia (*P* = 0.01), and history of cardiac diseases (*P* = 0.012) showed a significant relationship with HIS. In the cognitive function tests, both Korean version of the Mini-Mental State Examination and the Clinical Dementia Rating (Global and Sum of Boxes) had a significant relationship with HIS. Our study suggested HIS may have an association with some vascular factors and cognitive scales in community dwelling elderly. In this study, the HIS seemed to contribute to the evaluation of the quantity of vascular factors and to the prediction of status of cognitive function.

## 1. Introduction

The Hachinski ischemic score (HIS) is known to be a simple clinical tool, currently used for differentiating major types of dementia, such as primary degenerative, vascular or multi-infarct, and mixed type [1, 2]. Though there have been developments of newer tools for differentiating types of dementia, HIS has continuously and widely been used for such purpose [1, 3, 4]. There were many studies on the utility of HIS in the differentiation of dementia types, but investigations on the usefulness of HIS in the community dwelling elderly population are sparse.

Cerebrovascular factors are known to be the cause of vascular cognitive impairment and have negative effects on cognitive function [5, 6]. Cerebral infarction in itself, without the interaction with AD pathology, contributes to the likelihood of dementia [7]. When AD and IVD coexist as a mixed type of dementia, the cognitive symptom may be worse than when AD exists by itself [6]. Therefore vascular factors affect cognitive decline of both forms of dementia and it is important to assess vascular factors of elderly people. However, screening IVD or measuring the quantity of vascular risk factors of elderly people in a simple way is not established yet. Brain imaging techniques, such as CT, MRI, and MRA, are very good methods to assess cerebral vascular status and ischemic damages in brain. However, such methods are not cost effective as screening tools for a large population because of their high costs and low accessibilities.

High HIS is closely related with cerebrovascular disease and its vascular factors [8]. The composing items include history of hypertension and history of stroke as well as symptoms suggesting cerebral vascular events [2]. Therefore we thought HIS can be a useful screening tool representing the quantity of vascular factors in elderly community dwelling.

For a dementia patient, high HIS indicates high probability of IVD. Vascular cognitive impairment covers a continuous wide spectrum of cognitive dysfunction ranging from mild cognitive impairment of vascular origin to overt IVD [9, 10]. Therefore, even nondemented individuals could be on increased risk of IVD or mild cognitive impairment of vascular origin when their HIS is high. We also thought nondemented people with higher HIS have a higher rate of cognitive decline because vascular risk factors affect negatively cognition of individuals with vascular cognitive impairment as well as individuals with AD pathology [6, 11].

To evaluate such hypotheses, we investigated the relationship between HIS and vascular factors and the relationship between HIS and cognition in a community dwelling.

## 2. Methods

### 2.1. Study Participants

This study is an epidemiological survey. The source of the sample for this study was inhabitants aged 65 or over recorded in national residents registration lists within one defined rural geographic area of Goryeong Province, South Korea, in 2010. Participants were recruited through a community survey. The survey was announced by public leaders of each village of the Province and the people gathered in the community hall. Residents who did not come to the community hall were checked and a staff of regional public health center visited their home and encouraged them to participate in the survey. The residents aged 65 or over identified from registration lists constituted the population of 673 people. About 17% (114) did not live at the address. From the 559 real residents, 96 people refused the interview, or could not meet 73 people who went to work. Therefore, 392 (70% of real residents) participated in the survey. We excluded 44 people who did not complete the survey in analysis and 348 (62% of real residents) who completed interviews, blood tests, and neurological examinations were the sample size of this study.

All participants gave their written informed consent before participating in this study. The data collection protocol was approved by the Institutional Review Board of Daegu Catholic University Medical Center in Daegu, Korea.

### 2.2. Assessment and Measurements

The survey was carried out within one recruitment phase; five psychologists, 4 research nurses, and 2 neurologists were split into two survey teams. Each team also had administrative support of the regional public health centre of Goryeong Province. The people previously instructed to come in a fasting state and gathered in the community hall at the survey day. Residents who supposed to come to the community hall were checked of attendance. The staff of the regional public health center visited homes of each nonattendants and encouraged them to participate in the survey. The psychologists trained and supervised by the project neurologist carried out cognitive tests, and research nurses checked blood pressure, height, body weight, and head circumference and took disease histories and took blood samples. Neurologists, including a project manager, took neurological examinations and interviewed all the participants further about the cognitive function based on the result of cognitive tests and anthropological data, while also checking HIS. People who quit during the investigation were excluded from the statistical analysis of this study. Height and body weight were measured automatically by a machine and body mass index (BMI) was calculated by the results. Information was collected from participants and their caregivers, like spouses and children, and close friends living nearby family members, parental occupations, area of residence before age 20, duration of formal education, and literacy of the Korean alphabet, Hangul. Self-reported diagnoses of and treatment histories for diabetes, hypertension, hyperlipidemia, heart disease, stroke, depression, trauma, and other illnesses were recorded. Self-reported histories of alcohol drinking, smoking, and visual and auditory acuity and family histories of dementia and stroke were recorded also.

### 2.3. Cognitive Function Screening Tests and HIS

Cognitive function was assessed using the Korean version of the Mini-Mental State Examination (K-MMSE) [12] (rating 0–30, higher scores indicate good cognitive function) [13] and the Clinical Dementia Rating (CDR) (rating 0–5, lower scores indicate good cognitive function) scale including Clinical Dementia Rating Sum of Boxes (CDR-SB) [14] (rating 0–25, lower scores indicate good cognitive function) [15]. CDR was composed of six items, memory, orientation, judgment and problem solving, community affairs, home and hobbies, and personal care. Each item rated from 0 to 5. CDR 0 indicates normal and CDR 5 indicates severe dementia. Modified HIS by Rosen et al. [2] were also administered. This scoring system was composed of eight items, stepwise deterioration, somatic complaint, emotional incontinence, history of hypertension, history of strokes, abrupt onset, focal neurologic symptoms, and focal neurologic signs. The last four items received 2 points and the others received get 1 point which resulted in a total score of 12.

### 2.4. Statistical Analysis

Baseline analyses were carried out to determine the mean value and standard deviation of demographic characteristics including the presence of vascular factors and cognitive scores.

For the analysis of correlation between vascular factors and HIS, independent *t*-test was used. The *P* values are calculated by independent *t*-test between the mean HIS of participants with described factors and the mean HIS of participants without the described factors (Table 2).

We divided the sample to three groups according to K-MMSE scores and compared the HIS scores among the groups by ANOVA test. For the analysis of correlation between cognitive function and HIS, we also administered Pearson correlation coefficient. Simple linear regression analysis and multiple linear regression analysis were further performed to verify the results. Initial analyses were carried out to clarify vascular factors associated with HIS score. These associations were further analyzed dividing the subjects from low HIS score and to high HIS score and statistical evaluations on these subgroups were made with using independent *t*-test and Chi-square tests.

Data were expressed as mean ± standard deviation for continuous variables and frequency and percentage for categorical variables. Statistical significance was evaluated with a two-sided significance level of 0.05. All statistical analyses were performed by the IBM SPSS Statistics 19.0 (IBM Corp., Armonk, NY, USA).

## 3. Results

Baseline characteristics of the study participants were presented in Table 1. The mean age of the participants was 74.06 ± 5.51 years old and the male participants were about half of the female participants, reflecting the longevity of females in Korea. About one-third of the participants 127 (36.5%) had hypertension. The mean value of education was 2.79 ± 3.21 years which reflects their poor socioeconomic status during their school ages. The mean value of K-MMSE was 23.16 ± 4.73, the mean value of global CDR was 0.23 ± 0.28, and the mean value of CDR-SB was 0.56 ± 1.01.

Statistical analyses were performed to know the relation of each vascular factor and HIS. There were significant relationships between history of stroke (*P* < 0.001), history of hypertension (*P* = 0.008), family history of dementia (*P* = 0.01), and history of cardiac diseases, respectively (*P* = 0.012). Gender, family history of stroke, history of diabetes mellitus, and history of hyperlipidemia did not show a significant relationship with HIS (Table 2). We further defined the population for groups of values of the MMSE and the mean of HIS for each group (Table 3).

**Figure 1:**
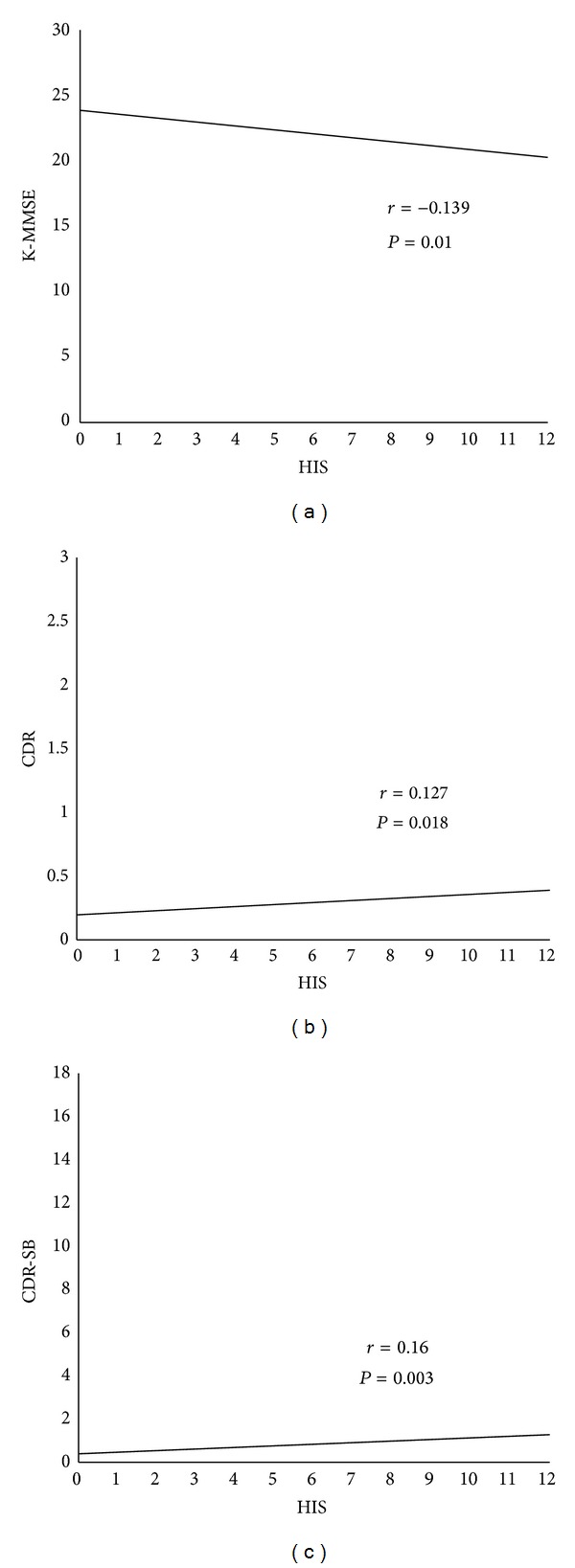
Relationship between HIS and cognitive function. (a) HIS and K-MMSE, (b) HIS and CDR, and (c) HIS and CDR-SB. Statistical analysis was made using Pearson correlation coefficient.

In the analysis of relationship between cognitive scales and HIS, K-MMSE, CDR, and CDR-SB showed significance relationship with HIS (Figure 1), which were further verified by simple linear regression analysis and multiple linear regression analysis. There was negative relationships between the HIS and K-MMSE score and positive relationships between HIS and CDR and CDR-SB.

In addition, we divided the study participants according to their HIS. One group was with HIS between 0 and 2 and the other group with HIS of 3–12 [2]. The analysis was performed on each variable in two groups. Both groups did not show any significant difference with age and sex. History of cardiac disease and history of stroke were higher in the group with HIS of 3–12 compared to the group with HIS of 0–2. Also the family history of dementia was higher in the group with HIS of 3–12. However, history of hypertension, history of diabetes, body mass index (BMI), history of hyperlipidemia, and family history of stroke did not have any significance (Table 4), which were further verified by simple linear regression analysis and multiple linear regression analysis. For scales that reflect cognitive dysfunction, such as K-MMSE, CDR, and CDR-SB, the group with HIS of 3–12 had significantly low K-MMSE and high CDR and CDR-SB compared to those of 0~2 group.

## 4. Discussion

Our study included a rural community dwelling aged 65 or over. Among the participants, the relationship between vascular factors, cognitive functions, and HIS was investigated. In the relationship between HIS and vascular factors, history of cardiac disease, history of stroke, family history of dementia, and history of hypertension were the significant correlation factors. In the correlation analyses between each cognitive function test and HIS, K-MMSE, CDR, and CD-SB showed relationship with HIS. K-MMSE showed negative relationship and the other two scale scores showed positive relationship.

The history of hypertension and history of stroke are included in checking items of HIS and that directly related with HIS. A history of hypertension and cardiac disease are important risk factors of cerebrovascular disease. In addition, these vascular factors not only related to high HIS but also related to vascular cognitive impairment [16–18]. Therefore, high HIS score may indicate the presence of more vascular factors which has an effect on the decrease in the cognitive functions [5, 6].

The relationship between family history of dementia and HIS is not reported yet as far as we know. In our study, the higher the HIS score, the higher the family history of dementia. The incidence of IVD is higher in Asian countries than in western countries [19, 20]. Therefore, we thought vascular factors of dementia are more related to the family history of dementia. As we know, vascular risk factors, such as hypertension, diabetes mellitus, and hyperlipidemia, could have some inherited traits. Moreover, the family members, including siblings and their parents, of the participants of this study had lived under a poor healthcare environment based in [21] lower socioeconomic classes before and after the Korean War which could guarantee poor control of many vascular factors of the family members. The rate of smoking in the area was high during the period and the hazard of chronic smoking of family members may also affect their descendants directly or indirectly. These factors may explain the relationship between HIS and family history of dementia. More investigation should be carried out to verify the relationship.

The relationship between HIS and dementia subtype of demented patient is relatively well known [22]. However, our study was done on the group of community dwelling elderly and most of the participants were not demented. Until now there was no known study of such associations on an elderly community sample. The reason why HIS shows relationship with vascular factors and cognitive scores of this community study may be explained as follows; first, the study participants might include people with cognitive impairment of vascular origin. The participants with higher HIS had more vascular factors prone to subcortical vascular dementia; therefore the possible vascular cognitive impairment led to a decrease in the cognitive function [21, 22]. Second, even in normal cognitive status, vascular factors represented as HIS may be directly related with cognitive decline. Third, the participants in our study were generally lowly educated which could have some effect on cognitive performance. In the previous investigation, the Korean with lower levels of education was more vulnerable to cognitive impairment with vascular factors [23]. The education of our sample was 2.79 ± 3.21 years and illiteracy rate was 23.6% (82 people).

Our study is unique because this is the first investigation done in the elderly community dwelling in Korea. The participants were based in one rural community with similar life styles which may help to achieve more meaningful results. We administered the CDR scale to get more reliable cognitive function [24] in addition to K-MMSE which has been a commonly used tool but has less sensitivity and specificity. The neuropsychological tests and HIS scoring were done more reliably by experienced neuropsychologists and neurologists, respectively.

However, our study has a few limitations. First, the participants included in our study could not exactly represent the entire elderly population aged 65 or over. We tried to include the whole population in the community but we did not randomly select the study participants. Therefore selection bias may be present. Secondly, this study is limited by the lack of pathology or brain imaging which can differentiate involvement of Alzheimer's disease or vascular cognitive impairment [9, 25, 26]. Third, this study was carried out in a community dwelling elderly where the demented participants would affect the results of our investigation though the demented rate was low. Generally, patients with vascular cognitive impairment have more vascular factors compared to AD and have higher HIS but it is difficult to state that the grade of cognitive dysfunction is worse than for AD.

For the conclusion, we provided the new possibility of using HIS in the community dwelling elderly population for the evaluation of the quantity of vascular factors. High HIS in elderly community dwelling may also suggest lower cognitive function especially for the participants with poorly controlled vascular factors. Further investigation with a larger sample and longitudinal study will verify such possibilities.

## Figures and Tables

**Table 1:** Demographic characteristics and vascular risk factors of subjects (*n* = 348).

Characteristics	Number (Percentage)
Age, yr (mean ± SD)	74.06 ± 5.51
Male (%)	112 (32.2)
Education years (mean ± SD)	2.79 ± 3.21
Family history of dementia (%)	19 (5.5)
Family history of stroke (%)	68 (19.5)
Diabetes mellitus (%)	51 (14.7)
Hypertension (%)	127 (36.5)
Hyperlipidemia (%)	18 (5.2)
Cardiac disease (%)	54 (15.5)
Stroke (%)	20 (5.7)
K-MMSE, mean ± SD	23.16 ± 4.73
CDR, mean ± SD	0.23 ± 0.28
CDR-SB, mean ± SD	0.56 ± 1.01
BMI, mean ± SD	22.87 ± 3.19

SD: standard deviation; K-MMSE, Korean version of Mini-Mental State Examination; CDR, Clinical Dementia Rating; SB, Sum of Boxes; BMI, body mass index.

**Table 2:** Relationship between HIS and vascular factors.

	HIS (mean ± SD)		*P* value∗
	Yes	No	
Male	2.52 (2.38)	2.23 (2.09)	0.251
Family history of dementia∗	3.58 (2.50)	2.25 (2.15)	0.01
Family history of stroke	2.51 (2.08)	2.27 (2.22)	0.419
Diabetes	2.69 (2.62)	2.26 (2.11)	0.199
Hypertension∗	2.73 (2.31)	2.09 (2.08)	0.008
Hyperlipidemia	3.00 (2.70)	2.28 (2.16)	0.178
Cardiac disease∗	3.15 (2.64)	2.17 (2.07)	0.012
Stroke∗	5.40 (3.22)	2.13 (1.97)	<0.001

HIS: Hachinski ischemic score; SD: standard deviation; ∗statistically significant with *P* < 0.05 by independent *t*-test.

**Table 3:** Relationship between HIS and the three K-MMSE groups according to K-MMSE scores.

	K-MMSE			*P* value
	0–17 (group 1)	18–24 (group 2)	25–30 (group 3)	
Number (percentage)	44 (12.64)	141 (40.52)	163 (46.8)	
HIS (mean ± SD)∗	2.93 (2.58)	2.48 (2.32)	2.02 (1.91)	<0.028

HIS: Hachinski ischemic score; K-MMSE: Korean version of Mini-Mental State Examination; SD: standard deviation; ∗statistically significant with *P* < 0.05 by ANOVA test.

**Table 4:** Relationship between HIS and cognitive function and HIS and vascular factors in two groups divided by HIS score.

	HIS 0~2 (*N* = 217)	HIS 3~12 (*N* = 131)	*P* value
Age, yr (mean ± SD)∗	73.92 ± 5.40	74.31 ± 5.72	0.388
Male (%)^†^	63 (29.0)	49 (37.4)	0.105
Family history of dementia (%)^†^	6 (2.8)	13 (9.9)	0.004
Family history of stroke (%)^†^	37 (17.1)	31 (23.7)	0.132
Diabetes mellitus (%)^†^	34 (15.7)	17 (13.0)	0.492
Hypertension (%)^†^	73 (33.6)	54 (41.2)	0.155
Hyperlipidemia (%)^†^	11 (5.1)	7 (5.3)	0.911
Cardiac disease (%)^†^	25 (11.5)	29 (22.1)	0.008
Stroke (%)^†^	5 (2.3)	15 (11.5)	<0.001
K-MMSE, mean ± SD∗	23.59 ± 4.42	22.43 ± 5.13	0.025
CDR, mean ± SD∗	0.20 ± 0.26	0.29 ± 0.31	0.008
CDR-SB, mean ± SD∗	0.43 ± 0.68	0.79 ± 1.37	0.006
BMI, mean ± SD∗	22.73 ± 3.31	23.10 ± 2.98	0.246

HIS: Hachinski ischemic score; SD: standard deviation; K-MMSE, Korean version of Mini-Mental State Examination; CDR, Clinical Dementia Rating; SB, Sum of Boxes; BMI, body mass index. ∗statistically significant with *P* < 0.05 by independent *t*-test and ^†^Chi-square test.
